# Development and validation of a radiologically-based nomogram for preoperative prediction of difficult laparoscopic cholecystectomy

**DOI:** 10.3389/fmed.2025.1561769

**Published:** 2025-04-22

**Authors:** Bo Zhu, Yingxin Wang, Zhenduo Zhang, Liwei Wang, Yashuai Ma, Ming Li

**Affiliations:** Department of General Surgery, Shijiazhuang People's Hospital, Shijiazhuang, China

**Keywords:** predictive model, laparoscopic cholecystectomy, difficult laparoscopic cholecystectomy, nomogram, risk factor

## Abstract

**Background:**

Preoperative prediction of difficult laparoscopic cholecystectomy (DLC) remains challenging, as intraoperative anatomical complexity significantly increases complication risks. Current studies have not reached consensus on definitive risk factors for DLC.

**Materials and methods:**

This retrospective study aimed to identify DLC risk factors and develop a predictive model. We analyzed clinical data from 265 patients undergoing laparoscopic cholecystectomy (LC) at the Department of General Surgery, Shijiazhuang People’s Hospital, between September 2022 and June 2024. Risk factors were explored through least absolute shrinkage and selection operator (LASSO) regression, multivariate analysis, and receiver operating characteristic (ROC) curves, with a nomogram constructed for prediction.

**Results:**

Among 265 eligible patients, four independent risk factors were identified: thickness of gallbladder wall (*p* = 0.0007), cystic duct length (*p* < 0.0001), cystic duct diameter (*p* < 0.0001), and gallbladder neck stones (*p* = 0.0002). The nomogram demonstrated strong predictive performance, with an area under the curve (AUC) of 0.915 in the training cohort and 0.842 in the validation cohort. Calibration curves indicated excellent model fit.

**Conclusion and discussion:**

The proposed predictive model integrating gallbladder neck stones, thickness of gallbladder wall, cystic duct length, and cystic duct diameter may assist surgeons in preoperative DLC risk stratification. Further validation through multicenter prospective studies is warranted.

## 1 Introduction

Laparoscopic cholecystectomy (LC) has become the gold-standard intervention for gallbladder pathologies. Characterized by minimally invasive access ports, reduced intraoperative tissue trauma, accelerated postoperative recovery, and shortened hospitalization duration, its technical merits have propelled widespread adoption since its inception. While LC procedural protocols have been refined over decades, intraoperative challenges persist, particularly during the critical phase of Calot’s triangle dissection. In cases with distorted anatomical architecture—often due to chronic inflammation, fibrotic adhesions, or aberrant vasculature—visualization and safe separation of the cystic duct-artery complex may be compromised, thereby heightening the likelihood of iatrogenic biliary tract injury or hemorrhagic complications. Moreover, difficult surgeries are associated with a higher likelihood of conversion to open surgery. According to the Tokyo Guidelines, open surgery is considered a reliable rescue procedure in the event of DLC (1, 2). Therefore, preoperative assessment of operative difficulty in LC is essential. A previous study developed an intraoperative gallbladder scoring system to predict the likelihood of conversion to open surgery (3). Other studies have focused on identifying risk factors for surgical challenges in cases of acute cholecystitis (4, 5). However, limited research exists on difficulties encountered during LC associated with chronic cholecystitis, gallbladder polyps, or other benign gallbladder pathologies. This study aims to collect clinical data from patients undergoing LC for gallbladder diseases, analyze risk factors for intraoperative difficulties, and develop a preoperative predictive model for challenging LC procedures. The goal is to provide surgeons with evidence-based guidance for risk evaluation, reduce complication rates, and improve clinical outcomes through optimized patient management.

## 2 Materials and methods

A retrospective analysis was conducted on 265 patients scheduled for laparoscopic cholecystectomy at the Department of General Surgery, Shijiazhuang People’s Hospital, between September 2022 and June 2024. Inclusion criteria were: (a) clear surgical indications for LC; (b) all procedures performed by surgeons with ≥10 years of general surgery experience; and (c) complete medical records. Exclusion criteria included: (a) operating on organs other than or beside LC; (b) history of prior upper abdominal surgery; and (c) confounding factors unrelated to LC (e.g., anesthesia complications, equipment malfunction). The study protocol was approved by the Ethics Committee of Shijiazhuang People’s Hospital (Ethics Review Certificate No.: [2025001]) and adhered to the Declaration of Helsinki.

Demographic data (age, sex, BMI), lifestyle habits (smoking status, alcohol consumption), laboratory parameters (total bilirubin, direct Bilirubin, aspartate aminotransferase, Alanine Aminotransferase, alkaline phosphatase, uric acid, creatinine, triglycerides, white blood cell count, hematocrit, fasting blood glucose), medical history (diabetes, coronary artery disease, hypertension), and imaging findings (gallbladder neck stone, thickness of gallbladder wall, cystic duct length, and cystic duct diameter) were collected. Surgical timing (whether LC was performed within 24 h of admission) and presence of sepsis were also recorded. Laboratory tests were conducted on admission after an overnight fast. All sepsis cases were secondary to cholecystitis and managed in general wards. Cystic duct dimensions (length and diameter) were measured via magnetic resonance cholangiopancreatography (MRCP). The final measurement was calculated as the average of three consensus readings obtained through blinded review by three independent radiologists.

The criteria for defining difficult laparoscopic cholecystectomy were as follows: (a) total operative time ≥120 min; (b) failure to achieve the Critical View of Safety (CVS) within 40 min; and (c) conversion to open surgery due to intraoperative technical challenges (e.g., severe adhesions, uncontrolled bleeding) or major complications (e.g., bile duct injury). Patients meeting any one criterion were assigned to the DLC group, whereas those fulfilling none were classified into the non-difficult laparoscopic cholecystectomy (NDLC) group (4).

Continuous variables were expressed as median with interquartile range after assessing normality using the Shapiro–Wilk test. Between-group comparisons were performed with the independent t-test (for normally distributed data) or Mann–Whitney U test (for non-normally distributed data). Categorical variables were presented as frequencies (%), and compared using Pearson’s chi-square test or Fisher’s exact test (for cell counts <5). The dataset was randomly split into a training cohort (*n* = 185, 70%) and a validation cohort (*n* = 80, 30%). No missing data required imputation due to complete clinical records.

First, LASSO regression was applied to the training cohort to screen potential predictors of difficult laparoscopic cholecystectomy. The optimal penalty parameter (*λ*) was determined through 10-fold cross-validation with minimum criteria, and features with non-zero coefficients were retained (6). Subsequently, these variables were entered into a multivariable logistic regression model, and predictors with *p* < 0.05 were used to construct a nomogram. Model performance was evaluated by calculating the AUC for discrimination and plotting calibration curves for accuracy. All analyses were conducted in R statistical software (version 4.2.2; R Foundation for Statistical Computing). A two-tailed *p*-value < 0.05 defined statistical significance.

## 3 Results

A retrospective study was conducted between September 2022 and June 2024, including 265 patients who underwent LC for gallbladder diseases. Among these, 102 patients were classified into the DLC group and 163 into the NDLC group. Comparative analyses of clinical characteristics between the two groups are summarized in Tables 1–4. The cohort was randomly divided into a training set (*n* = 185, 70%) and a validation set (*n* = 80, 30%) using a 7:3 allocation ratio. Normality tests were con-ducted for continuous variables, and the results are presented in Appendix Table 1. Comparison of differences between the training and validation sets is shown in Appendix Table 2. The results indicated that there were no significant differences in variables between the training and validation sets (*p* > 0.05).

**Table 1:** Comparison of general conditions between the NDLC group and the DLC group.

Clinical data	NDLC (*n* = 163)	DLC (*n* = 102)	*p* value
Gender			
Male	87 (53.4)	52 (51.0)	
Female	76 (46.6)	50 (49.0)	0.8
Age (year)	63 (49.5, 69.5)	60 (48, 69)	0.446
BMI (kg/m^2^)	25.2 (23.4, 28.0)	25.4 (23.1, 27.7)	0.95
Smoking			
Yes	44 (27.0)	30 (32.4)	
No	119 (73.0)	72 (70.6)	0.775
Alcohol			
Yes	39 (23.9)	33 (32.4)	
No	124 (76.1)	69 (67.6)	0.174

**Table 2:** Comparison of Laboratory Indicators Between the NDLC Group and the DLC Group.

Clinical Data	NDLC (*n* = 163)	DLC (*n* = 102)	*p* value
TBIL (μmol/L)	15.4 (11.0, 21.1)	19.9 (12.7, 30.8)	<0.001
DBIL (μmol/L)			
≤8	80 (49.1)	58 (56.9)	
>8	83 (50.9)	44 (43.1)	0.268
AST (U/L)			
≤40	32 (19.6)	26 (25.5)	
>40	131 (80.4)	76 (74.5)	0.332
ALP (U/L)	78 (62, 101)	82.5 (64.2, 110)	0.194
ALT (U/L)			
≤50	115 (70.6)	69 (67.6)	
>50	48 (29.4)	33 (32.4)	0.717
UA (μmol/L)			
≤428	103 (63.2)	55 (53.9)	
>428	60 (36.8)	47 (46.1)	0.171
Cr (μmol/L)	36 (53, 74)	64.5 (55,.2 75)	0.320
WBC (10^9/L)	6.5 (5.0, 9.15)	6.9 (5.5, 9.8)	0.142
HCT (%)	39.2 (36.5, 41.9)	40.3 (36.2, 42.7)	0.299
FPG (mmol/L)	5.5 (5.0, 6.8)	5.8 (5.1, 7.3)	0.190
TG (mmol/L)			
<2.26	141 (86.5)	86 (84.3)	
≥2.26	22 (13.5)	16 (15.7)	0.753

**Table 3:** Comparison of imaging indicators between the NDLC Group and the DLC group.

Clinical data	NDLC (*n* = 163)	DLC (*n* = 102)	*p* value
TOGW (mm)	3.0 (2.0, 3.7)	3.75 (3.0, 4.9)	<0.001
CDL (mm)	25 (20, 30)	17 (13, 20)	<0.001
CDD (mm)	4 (3.1, 5.1)	6 (4.0, 8.0)	<0.001
GNS			
Yes	31 (19.0)	50 (49.0)	
No	132 (81.0)	58 (51.0)	<0.001

**Table 4:** Comparison of medical history and other indicators between the NDLC group and the DLC group.

Clinical data	NDLC (*n* = 163)	DLC (*n* = 102)	*p* value
Surgery in 24 h			
Yes	74 (45.4)	42 (41.2)	
No	89 (54.6)	60 (58.8)	0.584
Sepsis			
Yes	8 (4.9)	8 (7.8)	
No	155 (95.1)	94 (92.2)	0.427
Hypertension			
No	83 (50.9)	64 (59.8)	
Grade 1	11 (6.7)	3 (2.9)	
Grade 2	29 (17.8)	14 (13.7)	
Grade 3	40 (24.5)	24 (23.5)	0.365
Coronary heart disease			
Yes	40 (24.5)	24 (23.5)	
No	123 (75.5)	76 (74.5)	0.978
Diabetes			
Yes	35 (21.5)	18 (17.6)	
No	128 (78.5)	84 (82.4)	0.549
Postoperative pathology			
Chronic cholecystitis	86 (52.8)	40 (39.2)	
Acute simple cholecystitis	44 (27.0)	32 (31.4)	
Acute suppurative cholecystitis or Gangrenous cholecystitis	21 (12.9)	23 (22.5)	
Gallbladder polyp	12 (7.3)	7 (6.9)	0.094

Univariate analysis of postoperative pathological findings revealed no significant differences in the distribution of acute simple cholecystitis, acute suppurative/gangrenous cholecystitis, chronic cholecystitis, and gallbladder polyps between the DLC and NDLC groups (*p* = 0.094). Since pathological diagnoses require postoperative confirmation and lack preoperative predictive utility, these parameters were excluded from the risk prediction model (Table 4).

**Figure 1:**
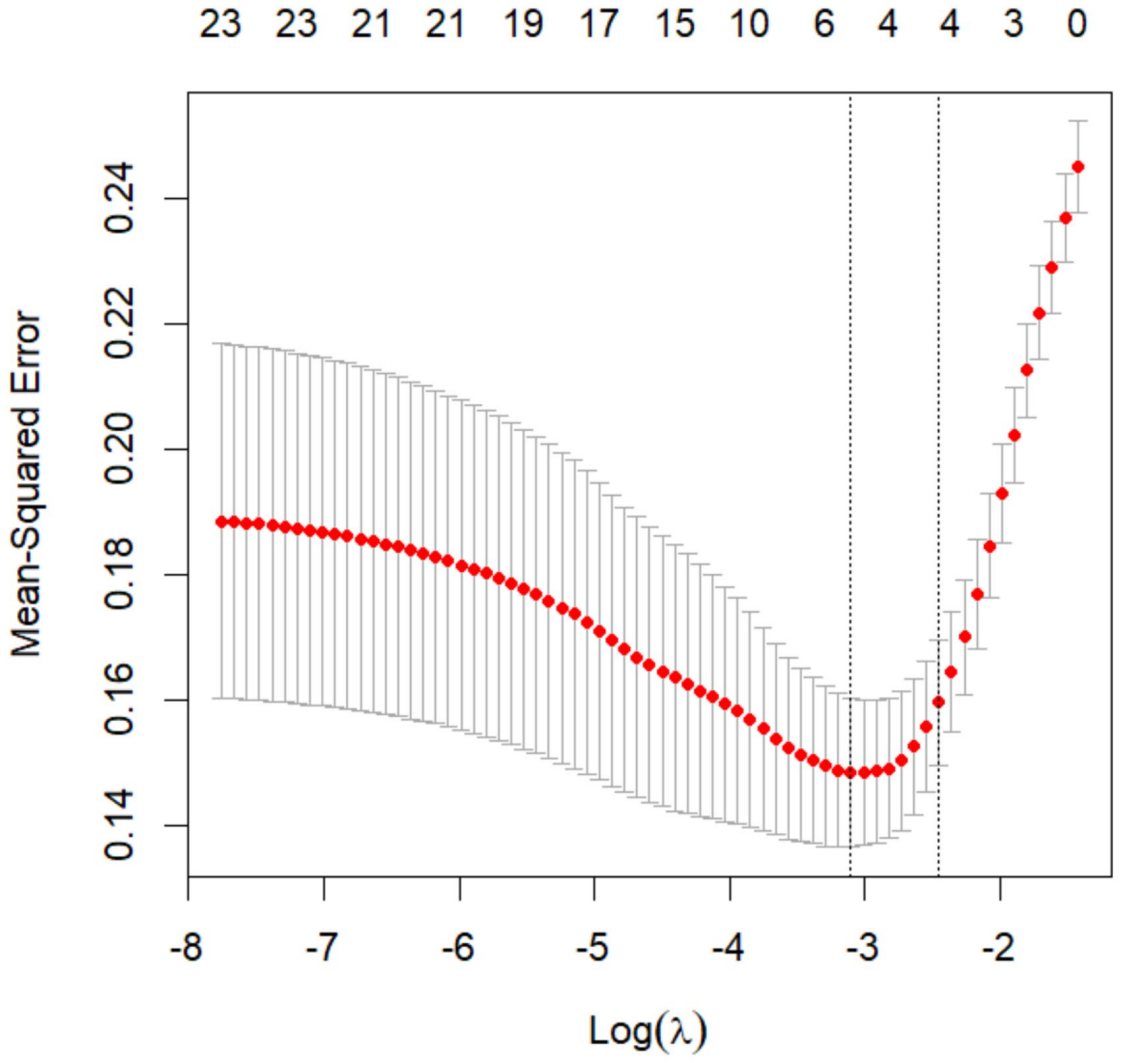
Lasso regression results.

**Figure 2:**
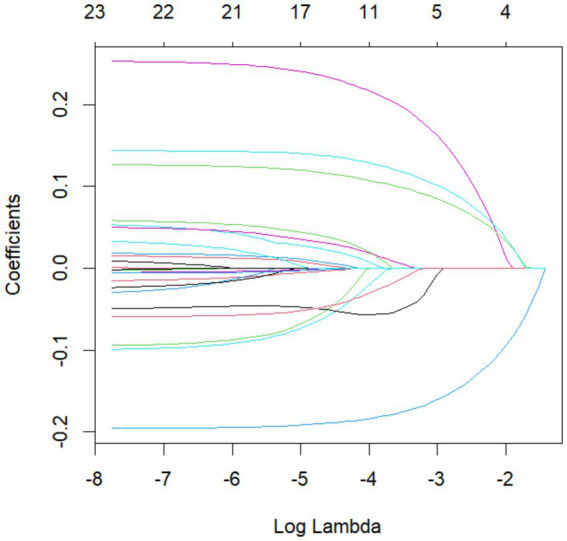
Lasso regression results.

In the LASSO regression model, potential predictors for DLC were identified based on features with non-zero coefficients (Figures 1, 2). The LASSO analysis revealed that a history of diabetes, impacted gallbladder neck stones (GNS), thickness of gallbladder wall (TOGW), cystic duct length (CDL), and cystic duct diameter (CDD) were associated with DLC risk. These variables were subsequently included in a multivariable logistic regression model using the “rms” package in R software (version 4.2.2). As shown in Table 5, impacted GNS (*p* = 0.0002), GWT (*p* = 0.0007), CDL (*p* < 0.001), and CDD (*p* < 0.001) remained independent predictors of DLC after adjustment for confounders.

**Table 5:** The prediction model with binary logistic regression.

	Coef	S.E.	Wald Z	Pr (>|Z|)
Diabetes	−1.0510	0.5755	−1.83	0.0678
GNS	1.8390	0.4986	3.69	0.0002
TOGW (mm)	0.6831	0.2023	3.38	0.0007
CDD (mm)	0.5119	0.1141	4.49	<0.0001
CDL (mm)	−0.2141	0.0379	−5.64	<0.0001

**Figure 3:**
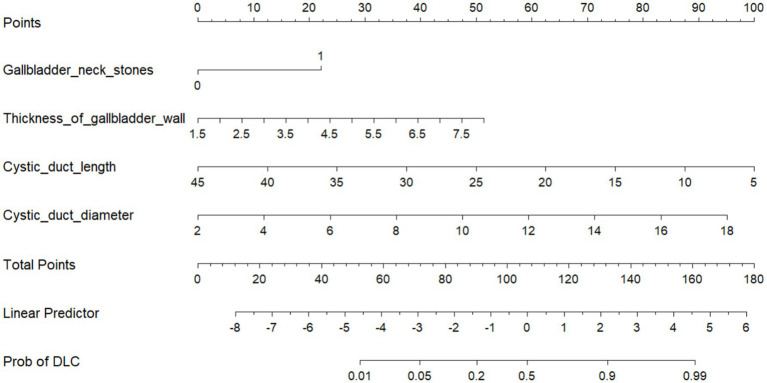
Nomogram.

Based on 10-fold cross-validation, LASSO regression was used to identify the optimal predictive variables for the model. A multivariable logistic regression model was established. Variance Inflation Factor (VIF) tests were performed, and all variables showed VIF values < 4, indicating no collinearity, and the model fit well. The final predictive model was constructed using variables with *p* values less than 0.05 in the multivariable logistic regression. These factors include GNS, TOGW, CDL, and CDD, as shown in Figure 3.

**Figure 4:**
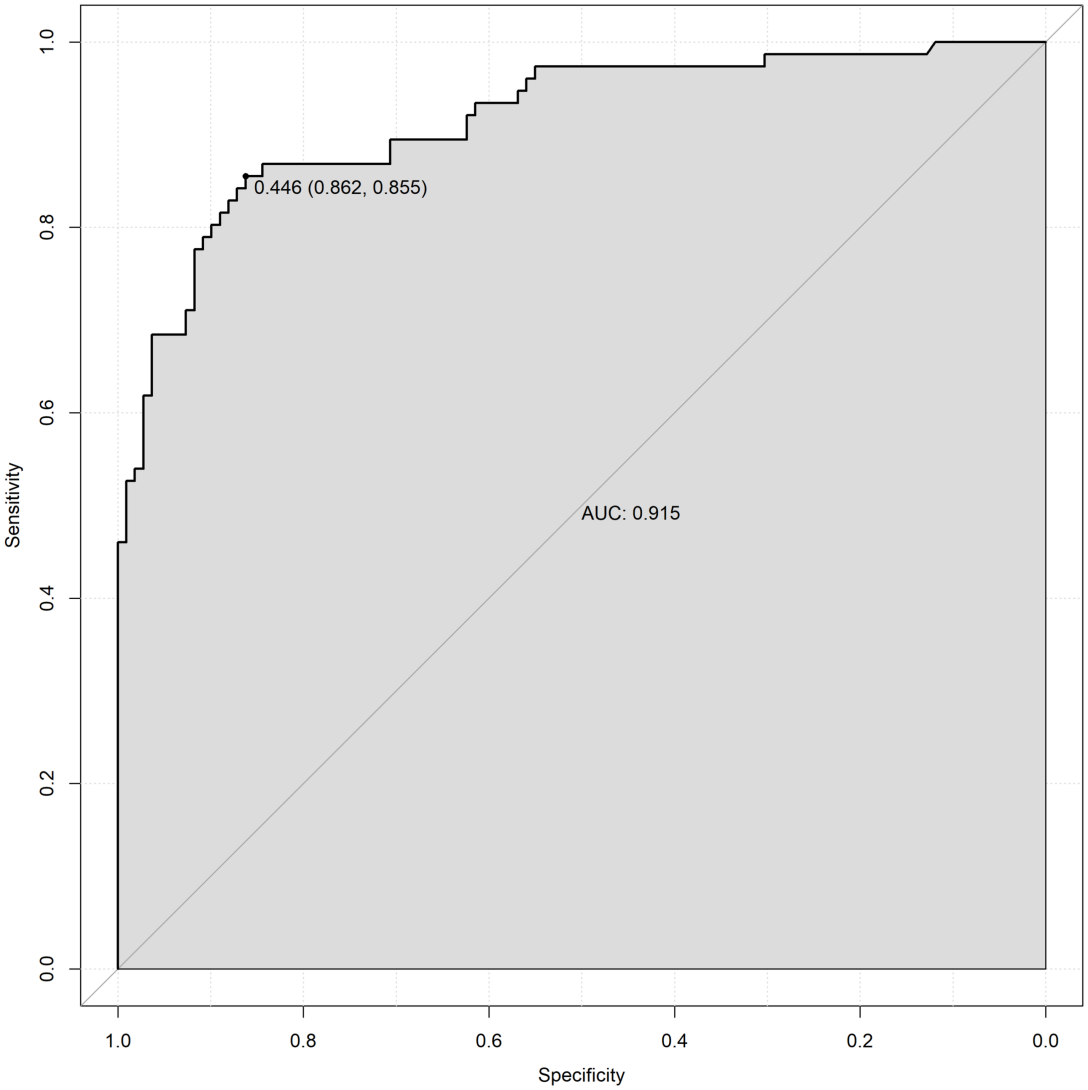
Training set ROC curve.

**Figure 5:**
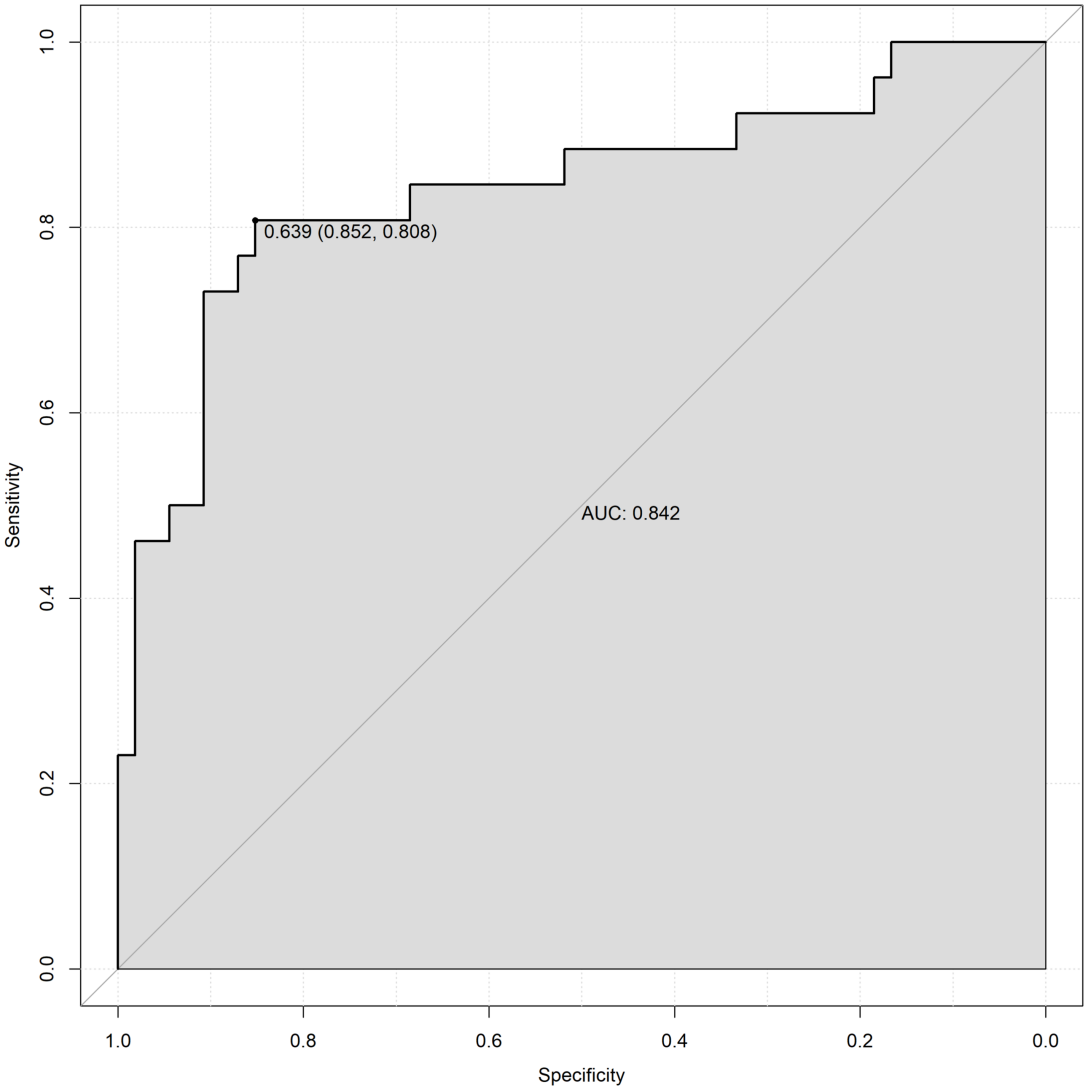
Validation set ROC curve.

**Figure 6:**
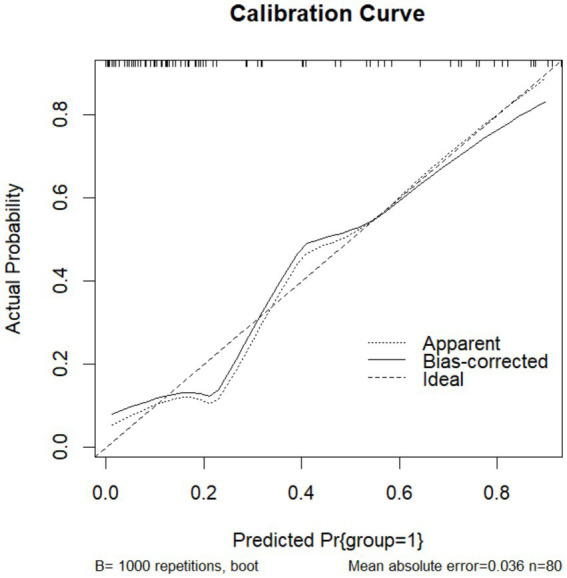
Training set calibration curve.

**Figure 7:**
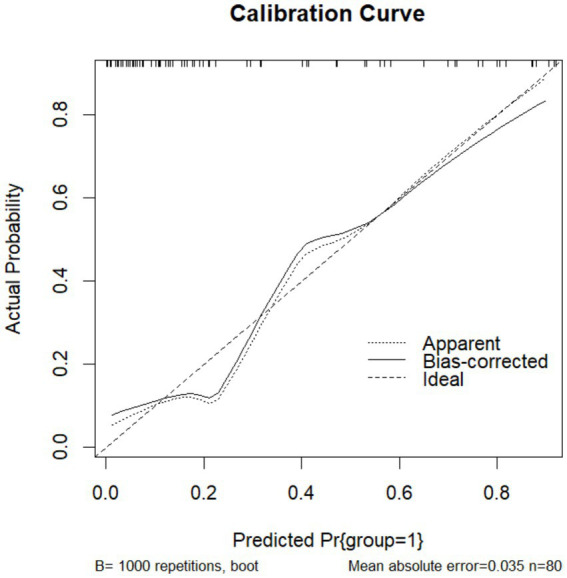
Validation set calibration curve.

To validate the prediction model, ROC curves were generated separately for the training and validation sets, and the AUC was calculated. A higher AUC value (closer to 1) indicates better predictive performance. The results are presented in Figures 4, 5. The AUC for the training set was 0.915 (95% CI: 0.873–0.958), and for the validation set, it was 0.842 (95% CI: 0.737–0.947). Calibration curves for both sets are shown in Figures 6, 7. The closer the calibration curve aligns with the reference line, the better the model’s prediction performance.

## 4 Discussion

Since 1985, LC has undergone decades of development, with both surgical instruments and techniques reaching maturity. The indications for LC have expanded significantly, establishing it as the first-line treatment for gallbladder diseases. Contraindications for LC may involve general patient conditions (surgical or anesthetic risks) or specific local factors, such as suspected gallbladder cancer, calcified gallbladder, gallbladder-enteric fistula, Mirizzi syndrome, or extensive prior upper abdominal surgery (5). Among LC complications, biliary and vascular injuries are the most common (7). In clinical practice, multiple factors may necessitate conversion to DLC (8). This study retrospectively analyzed preoperative laboratory tests and imaging findings in patients undergoing LC to evaluate statistical differences between DLC and NDLC, aiming to establish a preoperative prediction model for guiding surgical approach selection.

Regarding the definition of DLC, Spanish experts established a consensus in 2022 stating that bile duct injury, unclear anatomy, Mirizzi syndrome, severe inflammation in Calot’s triangle, conversion to open surgery, disease duration, sclerosing atrophic gallbladder, and pericholecystic abscess are predictive factors for cholecystectomy difficulty (9). This consensus serves as a critical foundation for surgical safety. However, most consensus indicators rely on intraoperative findings and thus cannot offer preoperative guidance to surgeons.

Previous studies have identified specific preoperative laboratory parameters as potential predictors of intraoperative difficulties, including white blood cell count, fibrinogen, C-reactive protein, and procalcitonin. These investigations primarily targeted patients with acute cholecystitis, demonstrating that such biomarkers effectively predict intraoperative challenges and offer surgical guidance (2, 4, 10). However, these biomarkers have limited utility in assessing intraoperative difficulty for patients undergoing laparoscopic cholecystectomy due to chronic cholecystitis or gallbladder polyps, as inflammatory markers (e.g., white blood cell count, C-reactive protein) typically remain within normal ranges in such populations. Consequently, this study focuses on imaging parameters including the thickness of gallbladder wall, cystic duct length, and cystic duct diameter.

Previous studies have categorized DLC based on parameters including operative time, intraoperative conversion, and complications (1, 11, 12). This study adopts the definition by Wu et al., where DLC is defined as: (a) total operative time >120 min; (b) failure to achieve CVS within 40 min; or (c) conversion to open surgery due to technical challenges or major complications (4). This study exclusively involved LC procedures performed by surgeons with >10 years of general surgical experience, whose case volumes far exceeded the established LC learning curve thresholds (rapid ascent phase: 30 cases; plateau phase: 250 cases), ensuring all operators were in the technical proficiency phase to minimize operator-dependent bias. Resident participation was restricted to laparoscopic camera assistance, a role proven by prior evidence to have negligible impact on operative time, thereby rigorously controlling for confounders related to surgical expertise. Additionally, CVS assessment was incorporated to reduce subjectivity in difficulty evaluation. As Calot’s triangle dissection poses the greatest technical challenge in LC, CVS provides an objective measure of this step’s complexity. This study relied solely on objective parameters for difficulty assessment, excluding subjective surgeon input.

The 2018 Tokyo Guidelines for Acute Cholecystitis and Acute Cholangitis recommend assessing the severity of acute cholecystitis using parameters such as local inflammation and ASA (American Society of Anesthesiologists) classification (2). However, this study did not collect ASA scores or acute cholecystitis severity grades, as our focus was specifically on intraoperative technical difficulty. The ASA classification reflects a patient’s overall anesthesia risk, which has limited relevance to the primary objective of evaluating intraoperative anatomical challenges. Instead, the parameter “surgery performed within 24 h of admission” was utilized to indirectly reflect the urgency of intervention. Furthermore, since our cohort included patients with acute or chronic cholecystitis and gallbladder polyps, the Tokyo Guidelines’ inflammatory grading system—primarily designed for acute cases—was not applied.

Numerous studies demonstrate that early surgery is effective for acute cholecystitis, while partial cholecystectomy or cholecystostomy should be considered for high-risk patients (2, 13–15). Recent research on DLC has primarily focused on acute cholecystitis (5, 11, 16, 17). In contrast, studies involving LC for chronic cholecystitis and gallbladder polyps remain limited. This study included all LC patients with gallbladder diseases to identify general predictors of DLC. Among 102 DLC and 163 non-DLC cases, baseline characteristics differed significantly between groups. After 7:3 training-validation split, LASSO regression on the training set identified DM, GNS, TOGW, CDL, and CDD as potential DLC predictors. To validate these findings, a multivariable logistic analysis was performed, which demonstrated that GNS (*p* = 0.0002), TOGW (*p* = 0.0007), CDL (*p* < 0.001), and CDD (*p* < 0.001) were independent risk factors for difficult laparoscopic cholecystectomy (DLC).

Tongyoo et al. developed a predictive model for DLC incorporating five clinical parameters (age >50 years, male sex, history of biliary inflammation, obesity, prior abdominal surgery) and four ultrasound features (impaired gallbladder contraction, gallbladder wall thickness ≥4 mm, pericholecystic fluid, impacted stones) (18). This differs from our methodology. In our analysis, LASSO regression was employed for feature selection due to its adaptability and ability to automatically retain the most significant predictors while excluding irrelevant variables, thereby improving model interpretability and clinical utility. Following LASSO regression, parameters such as sex, BMI, and age were excluded, indicating their negligible impact on our model.

A study by Serban D et al. showed that diabetic patients undergoing LC often experience more severe complications, longer hospital stays, and higher mortality rates. This may be attributed to diabetic neuropathy, impaired host response to infection, and various structural tissue damages resulting from prolonged exposure to hyperglycemia (19). In our multivariate analysis, our results indicated that diabetes (*p* = 0.0678) was not a risk factor for the occurrence of DLC, which contrasts with the findings of Serban D et al. This discrepancy may be attributed to the following factors: First, the impact of DM on surgical difficulty might be mediated indirectly through other variables (e.g., metabolic abnormalities) rather than acting as a direct independent risk factor. Second, differences in sample size or glycemic control levels of DM patients between our cohort and Serban D et al.’s study might exist. Future research should further explore the interaction effects between DM and other metabolic indicators and expand the sample size to validate its potential role in DLC.

Our study suggests that GNS is a predictive factor for DLC, as patients with GNS are more likely to experience conversion from LC to DLC. This finding is consistent with previous studies (1, 20–24). GNS can induce acute cholecystitis due to temporary or permanent obstruction of the cystic duct, which can lead to the release of hemolytic lecithin, triggering chemical reactions, and may also result in infection due to retrograde bacterial spread. These three factors often occur in combination (25, 26). In severe cases, they can even cause gallbladder perforation and gangrene. These factors undoubtedly increase the likelihood of DLC.

Our study also identifies TOGW as another significant predictive factor for DLC. TOGW is measured by computed tomography and ultrasound, with the measurement taken at the thickest portion of the gallbladder wall. In benign gallbladder diseases, TOGW typically presents as localized or diffuse intraluminal thickening, which is associated with inflammation (26). TOGW as a trigger for DLC has been confirmed in several studies (5, 16, 22, 23, 27–29).

The length and diameter of the cystic duct are unique indicators in this study. The gallbladder duct connects the gallbladder to the common bile duct, extending along the neck of the gallbladder and eventually merging with the hepatic duct to form the common bile duct (30). In clinical practice, we have observed that a reduced cystic duct length and an increased cystic duct diameter, often due to inflammation or other factors, increase the difficulty of the surgery. A cohort study has also indicated that during LC, encountering a short and wide cystic duct complicates the ligation of the cystic duct (31). The normal length of the cystic duct ranges from 20 to 40 mm, and its diameter is 3 to 4 mm. By analyzing the preoperative MRCP and CT results and applying three-dimensional reconstruction technology, we can obtain more accurate measurements. A shorter cystic duct makes it more challenging to expose CVS, whereas an enlarged diameter of the cystic duct complicates the ligation process. In some instances, an overly wide cystic duct precludes the use of absorbable clips for ligation. For such patients, we have to resort to laparoscopic suturing to close the cystic duct, which undoubtedly increases the surgical complexity, prolongs the operation time, and heightens the risk of complications.

A nomogram is a quantitative graphical tool that visually represents the functional relationships between variables through scaled axes and connecting line segments. By summing points assigned to each predictor, the total score estimates the probability of a clinical outcome (32). In this study, gallbladder neck stones, gallbladder wall thickness, cystic duct length, and cystic duct diameter were identified as key predictors of DLC. The nomogram-based model integrates these four parameters to quantify DLC risk. Model performance was validated through ROC curves and calibration plots, demonstrating excellent discriminative ability and alignment between predicted and observed outcomes. This tool provides individualized risk stratification with high clinical utility, enabling surgeons to preoperatively identify high-risk patients and optimize surgical planning for DLC.

This study innovatively incorporated the parameters of the CDL and CDD, and the developed model demonstrated good performance. As a single-center, retrospective study, it also has limitations. External validation was not conducted, and there was insufficient control of confounding factors. The statistical methods employed in this study can only address known confounders and cannot eliminate the potential influence of unknown variables.

## 5 Conclusion

In this study, the nomogram provides a good prediction for DLC. By analyzing preoperative clinical data, surgeons can gain an initial understanding of the potential difficulty of the surgery and make adequate preoperative preparations to manage the risk of DLC that may arise during the procedure. However, this study still requires an expansion of the sample size to reduce error, and further multicenter, prospective studies are needed to validate the findings.

## Data Availability

The raw data supporting the conclusions of this article will be made available by the authors, without undue reservation.

## Supplementary material

The Supplementary material for this article can befound online at: https://www.frontiersin.org/articles/10.3389/fmed.2025.1561769/full#supplementary-material

## Glossary

LC: Laparoscopic cholecystectomy
DLC: Difficult laparoscopic cholecystectomy
ROC: Receiver operating characteristic
AUC: Area under the curve
NDLC: Non-difficult laparoscopic cholecystectomy
TOGW: Thickness of gallbladder wall
CDL: Cystic duct length
CDD: Cystic duct diameter
GNS: Gallbladder neck stones
CVS: The critical view of safety
CT: Computed Tomography
MRCP: Magnetic Resonance Cholangiopancreatography
BMI: Body Mass Index
TBIL: Total Bilirubin
DBIL: Direct Bilirubin
ALT: Alanine Aminotransferase
AST: Aspartate Aminotransferase
ALP: Alkaline Phosphatase
UA: Uric Acid
Cr: Creatinine
WBC: White Blood Cell
HCT: Hematocrit
FPG: Fasting Plasma Glucose
TG: Triglycerides

## References

[1] Tongyoo A Chotiyasilp P Sriussadaporn E Limpavitayaporn P Mingmalairak C. The pre-operative predictive model for difficult elective laparoscopic cholecystectomy: a modification. Asian J Surg. (2021) 44:656–61. doi: 10.1016/j.asjsur.2020.11.018, PMID: 33349555

[2] Mayumi T Okamoto K Takada T Strasberg SM Solomkin JS Schlossberg D. Tokyo guidelines 2018: management bundles for acute cholangitis and cholecystitis. J Hepatobiliary Pancreat Sci. (2017) 25:96–100. doi: 10.1002/jhbp.519, PMID: 29090868

[3] Sugrue M Coccolini F Bucholc M Johnston A. Intra-operative gallbladder scoring predicts conversion of laparoscopic to open cholecystectomy: a WSES prospective collaborative study. World J Emerg Surg. (2019) 14:12. doi: 10.1186/s13017-019-0230-9, PMID: 30911325 PMC6417130

[4] Wu T Luo M Guo Y Bi J Guo Y Bao S. Role of procalcitonin as a predictor in difficult laparoscopic cholecystectomy for acute cholecystitis case: a retrospective study based on the TG18 criteria. Sci Rep. (2019) 9:10976. doi: 10.1038/s41598-019-47501-0, PMID: 31358829 PMC6662745

[5] Stoica PL Serban D Bratu DG Serboiu CS Costea DO Tribus LC. Predictive factors for difficult laparoscopic cholecystectomies in acute cholecystitis. Diagnostics. (2024) 14:346. doi: 10.3390/diagnostics14030346, PMID: 38337862 PMC10855974

[6] Bu F Deng X-h Zhan N-n Cheng H Wang Z-l Tang L. Development and validation of a risk prediction model for frailty in patients with diabetes. BMC Geriatr. (2023) 23:172. doi: 10.1186/s12877-023-03823-3, PMID: 36973658 PMC10045211

[7] Machado NO. Biliary complications post laparoscopic cholecystectomy: mechanism, preventive measures, and approach to management: a review. Diagn Therap Endoscopy. (2011) 2011:1–9. doi: 10.1155/2011/967017, PMID: 21822368 PMC3123967

[8] Sewefy AM Hassanen AM Atyia AM Gaafar AM. Retroinfundibular laparoscopic cholecystectomy versus standard laparoscopic cholecystectomy in difficult cases. Int J Surg. (2017) 43:75–80. doi: 10.1016/j.ijsu.2017.05.044, PMID: 28552812

[9] Manuel-Vázquez A Latorre-Fragua R Alcázar C Requena PM de la Plaza R Blanco Fernández G. Reaching a consensus on the definition of “difficult” cholecystectomy among Spanish experts. A Delphi project. A qualitative study. Int J Surg. (2022) 102:106649. doi: 10.1016/j.ijsu.2022.106649, PMID: 35525412

[10] Turhan VB. Komplike akut kolesistiti öngörmede preoperatif nötrofil/lenfosit ve trombosit/lenfosit oranları etkilidir. Turk J Trauma Emerg Surg. (2021). 471–476. doi: 10.14744/tjtes.2021.49956, PMID: 35485509 PMC10443129

[11] Di Buono G Romano G Galia M Amato G Maienza E Vernuccio F. Difficult laparoscopic cholecystectomy and preoperative predictive factors. Sci Rep. (2021) 11:2559. doi: 10.1038/s41598-021-81938-6, PMID: 33510220 PMC7844234

[12] Omiya K Hiramatsu K Shibata Y Fukaya M Fujii M Aoba T. Preoperative magnetic resonance cholangiopancreatography for detecting difficult laparoscopic cholecystectomy in acute cholecystitis. Diagnostics. (2021) 11:383. doi: 10.3390/diagnostics11030383, PMID: 33668281 PMC7996298

[13] Pisano M Allievi N Gurusamy K Borzellino G Cimbanassi S Boerna D. World Society of Emergency Surgery updated guidelines for the diagnosis and treatment of acute calculus cholecystitis. World J Emerg Surg. (2020) 15:61. doi: 10.1186/s13017-020-00336-x, PMID: 33153472 PMC7643471

[14] Gelbard R Karamanos E Teixeira PG Beale E Talving P Inaba K. Effect of delaying same-admission cholecystectomy on outcomes in patients with diabetes. J Br Surg. (2014) 101:74–8. doi: 10.1002/bjs.9382, PMID: 24338895

[15] Mou D Tesfasilassie T Hirji S Ashley SW. Advances in the management of acute cholecystitis. Annal Gastroenterol Surg. (2019) 3:247–53. doi: 10.1002/ags3.12240, PMID: 31131353 PMC6524093

[16] Gupta N Ranjan G Arora MP Goswami B Chaudhary P Kapur A. Validation of a scoring system to predict difficult laparoscopic cholecystectomy. Int J Surg. (2013) 11:1002–6. doi: 10.1016/j.ijsu.2013.05.037, PMID: 23751733

[17] Toro A Teodoro M Khan M Schembari E Di Saverio S Catena F. Subtotal cholecystectomy for difficult acute cholecystitis: how to finalize safely by laparoscopy—a systematic review. World J Emerg Surg. (2021) 16:45. doi: 10.1186/s13017-021-00392-x, PMID: 34496916 PMC8424983

[18] Tongyoo A Liwattanakun A Sriussadaporn E Limpavitayaporn P Mingmalairak C. The modification of a preoperative scoring system to predict difficult elective laparoscopic cholecystectomy. Journal of Laparoendoscopic & Advanced Surgical Techniques. (2023) 33:269–75. doi: 10.1089/lap.2022.0407, PMID: 36445743 PMC9997034

[19] Serban D Balasescu S Alius C Balalau C Sabau A Badiu C. Clinical and therapeutic features of acute cholecystitis in diabetic patients. Exp Ther Med. (2021) 22:10190. doi: 10.3892/etm.2021.10190, PMID: 34035855 PMC8135114

[20] Ramírez-Giraldo C Isaza-Restrepo A Conde Monroy D Castillo-Barbosa AC Rubio-Avilez JJ Van-Londoño I. What is the best score for predicting difficult laparoscopic cholecystectomy? A diagnostic trial study. Int J Surg. (2023) Publish Ahead of Print:1871–9. doi: 10.1097/JS9.0000000000000354, PMID: 37288543 PMC10389651

[21] Korayem IM Bessa SS. Preoperative predictors of difficult early laparoscopic cholecystectomy among patients with acute calculous cholecystitis in Egypt. BMC Surg. (2024) 24:329. doi: 10.1186/s12893-024-02532-x, PMID: 39449024 PMC11515539

[22] Veerank N Togale MD. Validation of a scoring system to predict difficult laparoscopic cholecystectomy: a one-year cross-sectional study. J West Afr College Surg. (2018) 8:23–39. PMID: 30899702 PMC6398510

[23] Singh S Agrawal N Khichy S. Preoperative prediction of difficult laparoscopic cholecystectomy: a scoring method. Nigerian. J Surg. (2015) 21:130. doi: 10.4103/1117-6806.162567, PMID: 26425067 PMC4566319

[24] Augustine A Vivek MAM Rao R. A comprehensive predictive scoring method for difficult laparoscopic cholecystectomy. Journal of minimal access. Surgery. (2014) 10:62. doi: 10.4103/0972-9941.129947, PMID: 24761077 PMC3996733

[25] Gutt C Schläfer S Lammert F. The treatment of gallstone disease. Dtsch Arztebl Int. (2020). 117:148–158. doi: 10.3238/arztebl.2020.0148, PMID: 32234195 PMC7132079

[26] Yu MH Kim YJ Park HS Jung SI. Benign gallbladder diseases: imaging techniques and tips for differentiating with malignant gallbladder diseases. World J Gastroenterol. (2020) 26:2967–86. doi: 10.3748/wjg.v26.i22.2967, PMID: 32587442 PMC7304100

[27] Nachnani J Supe A. Pre-operative prediction of difficult laparoscopic cholecystectomy using clinical and ultrasonographic parameters. Indian J Gastroenterol. (2005) 24:16–8. PMID: 15778520

[28] Bhandari TR Khan SA Jha JL. Prediction of difficult laparoscopic cholecystectomy: An observational study. Ann Med Surg. (2021) 72:103060. doi: 10.1016/j.amsu.2021.103060 PMC8591467 34815866

[29] Nidoni R. Predicting difficult laparoscopic cholecystectomy based on clinicoradiological assessment. J Clin Diagn Res. (2015). 9:PC09–12. doi: 10.7860/JCDR/2015/15593.6929, PMID: 26816942 PMC4717755

[30] Gong Y Wangzhe YC You F. The “hand as foot” teaching method in the anatomy of gallbladder. Asian J Surg. (2023) 46:1473–4. doi: 10.1016/j.asjsur.2022.09.027, PMID: 36229308

[31] Alyami R Alotaibi AE Alhoumaily B Momen I Aljanfawe H Algoblan M. Safety and efficacy of using stapler device for wide cystic duct ligation in acute setting of laparoscopic cholecystectomy. Sci Rep. (2024) 14:25062. doi: 10.1038/s41598-024-75398-x, PMID: 39443523 PMC11499593

[32] Zhang M Ding C Xu L Feng S Ling Y Guo J. A nomogram to predict risk of lymph node metastasis in early gastric cancer. Sci Rep. (2021) 11:22873. doi: 10.1038/s41598-021-02305-z, PMID: 34819570 PMC8613278

