# Supplementary material for: Development and validation of a radiologically-based nomogram for preoperative prediction of difficult laparoscopic cholecystectomy
Source: Front Med (Lausanne). 2025 Apr 22;12:1561769. doi: 10.3389/fmed.2025.1561769 (PMC12060169; doi:10.3389/fmed.2025.1561769)
Supplement: Supplementary file 1 [file Table_1.docx]

Supplementary Material

# Supplementary Data

Supplementary Material should be uploaded separately on submission. Please include any supplementary data, figures and/or tables.

Supplementary material is not typeset so please ensure that all information is clearly presented, the appropriate caption is included in the file and not in the manuscript, and that the style conforms to the rest of the article.

# Supplementary Figures and Tables

For more information on Supplementary Material and for details on the different file types accepted, please see [here](https://www.frontiersin.org/guidelines/author-guidelines#supplementary-material).

Appendix Table 1 Shapiro-Wilk normality test

| Data | W | P-value |
| --- | --- | --- |
| Age | 0.97354 | 7.863e-05 |
| TBIL | 0.45561 | < 2.2e-16 |
| ALP | 0.61181 | < 2.2e-16 |
| WBC | 0.86036 | 9.263e-15 |
| HCT | 0.8083 | < 2.2e-16 |
| FPG | 0.75585 | < 2.2e-16 |
| TOGW | 0.89742 | 1.948e-12 |
| CDL | 0.98395 | 0.004495 |
| CDD | 0.88371 | 2.354e-13 |
| BMI | 0.99115 | 0.1105 |
| Cr | 0.37382 | < 2.2e-16 |

Appendix Table 2 Comparison of Differences Between Training and Validation Sets

| Data | P-value |
| --- | --- |
| Gender | 0.3432 |
| Age | 0.3892130 |
| BMI | 0.2966 |
| Smoking | 0.5832 |
| Alcohol | 0.9434 |
| TBIL | 0.7171397 |
| AST | 1 |
| ALP | 0.8827014 |
| UA | 0.9569 |
| Cr | 0.9318056 |
| WBC | 0.8279037 |
| HCT | 0.3083023 |
| FPG | 0.6503332 |
| TOGW | 0.6669969 |
| CDL | 0.8785050 |
| CDD | 0.3219064 |
| TG | 0.1294 |
| Surgery in 24 hours | 0.3478 |
| Sepsis | 1 |
| Hypertension | 0.5727 |
| Coronary heart disease | 0.2893 |
| GNS | 0.3911 |
| Diabetes | 0.2417 |

## Supplementary Figures

**
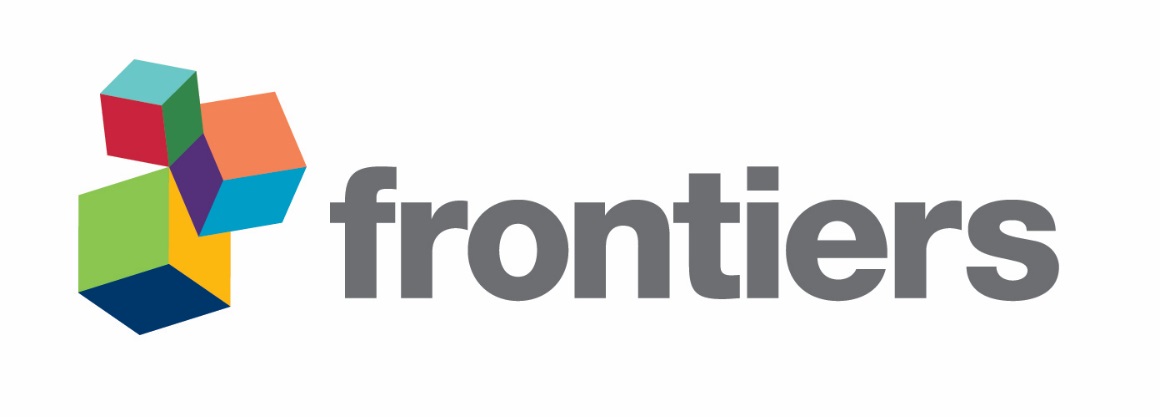
**

**Supplementary Figure 1.** The figure legends are required to have the same font as the main text, 12 point normal Times New Roman, single spaced. Please use a single paragraph for each legend and prepare the figures keeping in mind the PDF layout.
